# Dynamic Monitoring of Immunoinflammatory Response Identifies Immunoswitching Characteristics of Severe Acute Pancreatitis in Rats

**DOI:** 10.3389/fimmu.2022.876168

**Published:** 2022-05-19

**Authors:** Qian Zhuang, Liqiang Huang, Yue Zeng, Xu Wu, Gan Qiao, Minghua Liu, Lulu Wang, Yejiang Zhou, Yuxia Xiong

**Affiliations:** ^1^Department of Pharmacology, School of Pharmacy, Southwest Medical University, Luzhou, China; ^2^Institute for Clinical Trials of Drugs, Second People’s Hospital of Yibin, Yibin, China; ^3^Department of Gastrointestinal Surgery, Affiliated Hospital of Southwest Medical University, Luzhou, China

**Keywords:** severe acute pancreatitis, intestinal mucosal immune function, inflammation, immunosuppression, immunoswitching

## Abstract

**Background:**

Immune dysfunction is the main characteristic of severe acute pancreatitis (SAP), and the timing of immune regulation has become a major challenge for SAP treatment. Previous reports about the time point at which the immune status of SAP changed from excessive inflammatory response to immunosuppression (hypo-inflammatory response) are conflicting.

**Purposes:**

The aims of this study are to explore the immunological dynamic changes in SAP rats from the perspective of intestinal mucosal immune function, and to determine the immunoswitching point from excessive inflammatory response to immunosuppression.

**Methods:**

Retrograde injection of sodium taurocholate into the pancreaticobiliary duct was applied to establish a SAP model in rats. The survival rate and the activities of serum amylase and pancreatic lipase in SAP rats were measured at different time points after model construction. The pathological changes in the pancreas and small intestines were analyzed, and the levels of intestinal pro- and anti-inflammatory cytokines and the numbers of intestinal macrophages, dendritic cells, Th1, Th2, and T regulatory cells were assessed. Meanwhile, the SAP rats were challenged with *Pseudomonas aeruginosa* (PA) strains to simulate a second hit, and the levels of intestinal inflammatory cytokines and the numbers of immune cells were analyzed to confirm the immunoswitching point.

**Results:**

The time periods of 12–24 h and 48–72 h were the two death peaks in SAP rats. The pancreas of SAP rats showed self-limiting pathological changes, and the switching period of intestinal cytokines, and innate and adaptive immunity indexes occurred at 24–48 h. It was further confirmed that 48 h after SAP model construction was the immunoswitching point from excessive inflammatory response to immunosuppression.

**Conclusion:**

The SAP rats showed characteristics of intestinal mucosal immune dysfunction after model construction, and the 48th h was identified as the immunoswitching point from excessive inflammatory response to immunosuppression. The results are of great significance for optimizing the timing of SAP immune regulation.

## Introduction

Severe acute pancreatitis (SAP) is an acute abdominal disease with a high mortality. Immune dysfunction has been recognized as the predominant cause leading to severe SAP and SAP-related death (1–3). The immune response of SAP is characterized by excessive inflammatory response in the early stage, during which inflammation damages both tissues and immune cells. The pro-inflammatory and anti-inflammatory responses are counterbalanced in the middle stage. Along with increased inflammatory damage, immune function is decreased and eventually developed into immunosuppression (4). Immunomodulatory therapies *via* anti-inflammation and immuno-stimulation or both have been suggested as promising treatment of SAP, which can modulate the uncontrolled inflammatory response, reduce organ damage, and ameliorate prognosis of SAP patients. Notably, as pro-inflammatory and anti-inflammatory responses simultaneously occur in SAP (5), the timing of anti-inflammatory or immune-stimulatory intervention is an important factor for successful SAP treatment. Therefore, it is of much significance to monitor the immune status of SAP patients in order to determine the optimal timing of treatment.

The intestine as the largest immune organ and bacterial repository in the body is one of the most vulnerable target organs of SAP (6). During SAP, the excessive release of inflammatory cytokines and the impairment of intestinal microcirculation accompanied by massive apoptosis of immune cells lead to the inhibition of intestinal mucosal immune function and the injury of intestinal mucosal barrier (7–9). Uncontrolled inflammatory response and excessive apoptosis of intestinal immune cells are the main characteristics of mucosal immune dysfunction in SAP (10). Gut-derived endotoxemia and secondary infection due to intestinal bacterial translocation are accepted as important contributors towards increased mortality in SAP patients (11, 12). It is well acknowledged that the severity of SAP is positively correlated with mucosal immune dysfunction (10).

Cytokine level is one of the most essential indicators reflecting the immune status of SAP patients (2). Gut-derived endotoxins and cytokines mainly enter the systemic circulation through the intestinal lymphatic pathway and induce systemic inflammatory response, while blocking the mesenteric lymphatic vessels can reduce the systemic inflammatory response (13). Therefore, there is a positive correlation between intestinal and systemic inflammation through the intestinal–lymphatic pathway. However, few studies have explored the dynamic changes in intestinal mucosal immune function and inflammatory response during SAP.

In order to reveal the dynamic changes in intestinal mucosal immune function during SAP, the levels of intestinal pro- and anti-inflammatory cytokines and the numbers of innate and adaptive immune cells were monitored and analyzed in SAP rats at different time points after model construction to preliminarily determine the immunoswitching period. Moreover, *Pseudomonas aeruginosa* (PA) is one of the main pathogens of later systemic infectious complications of SAP, and has been applied to the study of sepsis-induced immunosuppression in a mouse model (14–16). Hence, the immunoswitching point was confirmed in SAP rats with a second hit of PA, which would provide reference for the research on the optimal timing of subsequent immunomodulatory drug intervention.

## Materials and Methods

### Reagents and Kits

Amylase, lipase, D-lactate, soluble tumor necrosis factor-α receptor (sTNF-αR), and secretory immunoglobulin A (sIgA) enzyme-linked immunosorbent assay (ELISA) kits were purchased from Nanjing Jiancheng Biological Engineering Institute (Nanjing, China). The chromogenic limulus amebocyte lysate kit was purchased from Bioendo Technology Co., Ltd (Xiamen, China). Rat interleukin-1beta (IL-1β) and transforming growth factor-β (TGF-β) ELISA kits were purchased from ExCell Biotech Co., Ltd (Shanghai, China). Rat TNF-α and IL-10 ELISA kits were purchased from Cusabio Biotech Co., Ltd (Wuhan, China). Rat IL-6 and IL-18 ELISA kits were purchased from Elabscience Biotech Co., Ltd (Wuhan, China). The rat IL-8 ELISA kit was purchased from Neobioscience Technology Co., Ltd (Shenzhen, China). The rat IL-4 ELISA kit was purchased from Invitrogen (Carlsbad, CA, USA). The specific antibodies against FITC anti-rat CD4, Alexa fluor^®^647, anti-rat IFN-γ, PE anti-rat IL-4, and Alexa fluor^®^ 647 anti-rat Foxp3 were purchased from BioLegend (San Diego, CA, USA). Rabbit anti-rat CD68 and rabbit anti-rat CD103 were purchased from Abcam (Cambridge, MA, UK). PE anti-rat CD25 was purchased from BD Biosciences (San Jose, CA, USA). The sources of other reagents were indicated in the specified methods.

### Animals

All animal experiments were conducted strictly in accordance with the guidelines and regulations of Southwest Medical University and preapproved by the Animal Ethics Committee of Southwest Medical University (approval numbers: 20180309063, Luzhou, China). The specific pathogen-free grade Sprague–Dawley rats (male, 6 weeks old, weight 200 ± 220 g) were purchased from the Dossy Experimental Animals Co., Ltd (permit number: SCXK 2013-24, Chengdu, China). The rats were housed in a specific pathogen-free environment (23 ± 1°C, 40%–70% relative humidity, and 12 h/12 h light–dark cycle), with free access to water and standard rodent diet.

### Experimental Protocol

The rats were randomly assigned to three groups: Normal group, Sham group, and SAP group. The Sham group and the SAP group were allocated into 11 subgroups according to time points of 1, 3, 6, 12, 24, 36, 48, 72, 120, 168, and 336 h after surgery. Rats were fasted for 12 h and anesthetized with 2.0% sodium pentobarbital (Sinopharm Chemical Reagent Co., Ltd., China). The animal model was established according to existing studies (17, 18). In brief, an abdominal incision was made along the midline to expose the biliopancreatic duct, and the liver hilum was temporarily closed with a microvascular clamp. Then, the biliopancreatic duct was cannulated using an intravenous indwelling trocar (Fenglin Medical Devices Co., Ltd., Jiangxi, China) with a catheter through the duodenum. The SAP rat model was established by a retrograde injection of 3.5% sodium taurocholate (Sigma-Aldrich, St. Louis, MO, USA) into the biliopancreatic duct using a micro-infusion pump. The microvascular clamp and intravenous indwelling trocar were removed 3 min later, and the abdominal incision was sutured. Postoperatively, 5 ml of normal saline was subcutaneously injected to compensate for fluid loss. The Sham group was injected with equal doses of normal saline, and the Normal group was not treated. Rats in each group were sacrificed at the above indicated time points. For survival analysis, the survival rate was recorded for 14 days after surgery.

**Figure 1:**
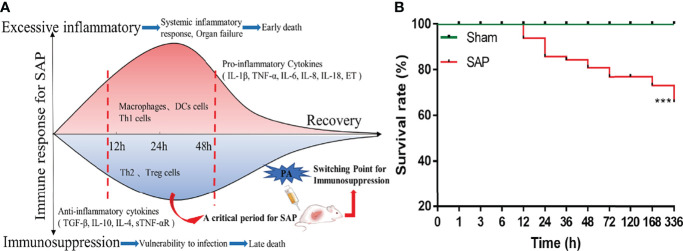
Flowchart of experimental schemes and survival situation of SAP rats. **(A)** Flowchart presenting the experimental schemes of this study. **(B)** The 14-day cumulative survival rate of rats. Differences are statistically analyzed using the Log-rank test (*n* = 10). ***p < 0.001 compared with the Sham group.

The blood samples were obtained through the abdominal aorta, kept at room temperature for 10 min, and centrifuged at 2,500 rpm for 15 min to collect serum, which was then stored at −80°C until further analysis. The pancreas and small intestines were harvested, washed in ice-cold normal saline, and stored at −80°C for further analysis. A small piece of pancreas and small intestines were fixed in 4% paraformaldehyde for pathological analysis. The mesenteric lymph nodes (MLNs) were harvested and gently ground with the inner core of a 5-ml syringe to make it pass through the 40-μm cell strainer to prepare a single-cell suspension of MLNs. Centrifugation was conducted at 1,000 rpm for 10 min, and the supernatant was discarded. Cell pellets of MLNs were resuspended in complete medium, and the concentration was adjusted to 10^6^/ml by cell counting plate for flow cytometry. An experiment flowchart is shown in **Figure 1A**.

### Pathological Observation

Pancreas and small intestines were fixed in 4% paraformaldehyde, dehydrated, and embedded in paraffin. Slices (5 µm) of the paraffin-embedded tissue were stained with hematoxylin and eosin. All the slices were graded by two experienced pathologists who were blind to the experimental protocol under a light microscope (Olympus Corporation, Tokyo, Japan). Inflammation, edema, hemorrhage, and acinar cell necrosis of pancreas were scored according to Schmidt’s standard (19). The severity of the small intestines was assessed as previously described by Chiu (20).

### Serum Amylase and Pancreatic Lipase Analysis

The activities of serum amylase and pancreatic lipase were determined with the corresponding assay kits according to the manufacturer’s instructions.

### Serum D-Lactate, Endotoxin, and Intestinal Cytokine Analysis

The levels of serum D-lactate and endotoxin were assayed using the commercially available detection kits, and the levels of pro- and anti-inflammatory cytokines including IL-1β, TNF-α, IL-6, IL-8, IL-18, TGF-β, IL-10, IL-4, and sTNF-αR in the small intestines were quantified using the relevant ELISA kits according to the manufacturer’s instructions. Absorption at 450 nm was examined using an Epoch 2 microplate reader (Bio-Tek Instruments, Winooski, VT, USA).

### Immunohistochemical Staining

Immunohistochemical staining was performed to determine the expressions of intestinal CD68 and CD103 to observe the changes in intestinal macrophages and dendritic cells. The paraffin-embedded slices were dewaxed, rehydrated, and treated for antigen retrieval according to standard procedures. Endogenous peroxidase was blocked by 3% hydrogen peroxide. After blocking the nonspecific antigens, the slices were incubated overnight at 4°C with anti-rat CD68 antibody or anti-rat CD103 antibody, followed by incubation with polymer adjuvant and horseradish peroxidase-labeled anti-rabbit IgG polymer (Bioss Biotech Co., Ltd., Beijing, China). Finally, the slices were stained with DAB (Beyotime Biotech Co., Ltd., Shanghai, China), and nuclei were counterstained with hematoxylin (Solarbio Technology Co., Ltd., Beijing, China). The samples were observed, and pictured under a Leica DMi8 inverted microscope (Leica Microsystem Ltd., Wetzlar, Germany). Images were analyzed using ImageJ software (National Institutes of Health, Bethesda, MD, USA).

### Th1/Th2 Cells Analysis

MLN cells were placed in a sterile 6-well plate with complete medium, and cultured with cell activation cocktail (BioLegend, San Diego, CA, USA) for 5 h in an incubator (Thermo Scientific, Waltham, MA, USA) with 5% CO_2_, at 37°C. After culturing for 2 h with brefeldin A solution (BioLegend), the cells were collected, centrifuged at 1,000 rpm for 5 min, and resuspended in PBS. Next, the cells were incubated with FITC anti-rat CD4 antibody for 15 min at room temperature in darkness, and then washed twice with PBS. Stained cells were fixed with fixation buffer (BioLegend) for 20 min at room temperature and washed twice with PBS. For intracellular staining of IFN-γ and IL-4, fixed cells were permeabilized using a permeabilization buffer (BioLegend) for 20 min at room temperature and incubated with Alexa fluor 647^®^ anti-rat IFN-γ antibody or PE anti-rat IL-4 antibody for 15 min at room temperature in darkness. After washing twice with cell staining buffer (BioLegend), the cells were suspended in cell staining buffer and the ratio of Th1/Th2 was determined by a FACSCalibur flow cytometer (BD Biosciences, San Diego, CA, USA) as previously described (21).

### T Regulatory Cells Analysis

The expression of Tregs from the MLNs was detected by a flow cytometer after staining with anti-rat-specific Abs conjugated with FITC, PE, and Alexa fluor 647^®^. To detect the proportion of CD4^+^ T cells in MLN cells and CD25 expression on the surface of CD4^+^ T cells, the cells were stained with FITC anti-rat CD4 antibody and PE anti-rat CD25 antibody for 30 min at room temperature. Simultaneously, for the measurement of intranuclear Foxp3, stained cells were washed with PBS and reacted with true-nuclear™ 1× fix working solution (BioLegend) for 60 min at room temperature. After washing twice with true-nuclear™ 1× perm buffer (BioLegend), the cells were stained with Alexa fluor 647^®^ anti-rat Foxp3 antibody for 30 min. After washing, the cells were suspended in cell staining buffer (BioLegend) and analyzed by a FACSCalibur flow cytometer (BD Biosciences) as previously described (22).

### Determination of the Immunoswitching Point From Excessive Inflammatory Response to Immunosuppression in SAP Rats

Rats were randomly assigned to four groups: Sham group, Sham + PA group, SAP group, and SAP + PA group. The Sham + PA group and the SAP + PA group were injected with 2.0×10^8^ CFU/kg of PA *via* the tail vein at 0, 12, 24, 36, 48, and 72 h after Sham or SAP construction, and the other groups were injected with equal doses of normal saline. Rats were sacrificed at 2 h after injection, and small intestines were collected. The levels of intestinal inflammatory cytokines were measured according to the manufacturer’s instructions. Meanwhile, the numbers of macrophages and dendritic cells were analyzed as previously described.

### Statistical Analysis

Data were analyzed by GraphPrism 8.0 software (San Diego, CA, USA) and expressed as mean ± standard deviation (SD). The comparison was conducted by Student’s *t*-test between two groups, and the comparison between multiple groups (including two factors with different time and model processing) was conducted using two-way analysis of variance (two-ANOVA). Counting data were tested by the chi-square test. The survival curves were analyzed using the Log-rank test. *p* < 0.05 was considered statistically significant.

## Results

### Death Peaks of SAP Rats Were Observed at 12–24 h and 48–72 h

There was no dead rat in the Sham group and Normal group, while the rats in the SAP group developed drowsiness, abdominal pain, and distension, and started to die at 12 h after model construction. A total of 11 rats died at 12–24 h and 5 rats died at 48–72 h. The 14-day cumulative survival rate of SAP rats was 65.72% (**Supplemental Table 1**; **Figure 1B**). The mortality rule of SAP rats was basically consistent with previous reports (23, 24). The results suggested that 12–24 h and 48–72 h were the two death peaks of SAP rats.

### The Pancreas of SAP Rats Presented the Self-Limiting Pathological Changes

To further clarify whether the SAP rat model was successfully established, we observed the pathological changes in pancreas, and measured the activities of serum amylase and pancreatic lipase.

**Figure 2:**
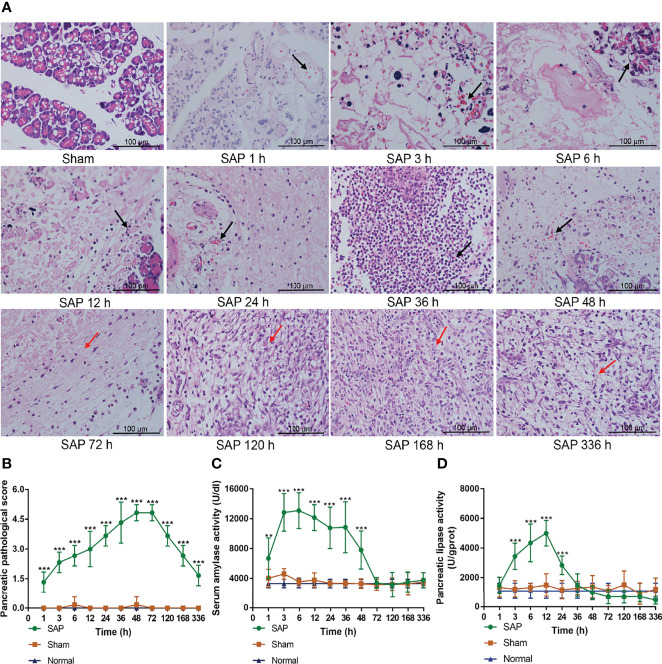
Damage changes in the pancreas at different time points. **(A)** Pathological observation of pancreas (*n* = 3,200 × magnification, scale bar = 100 µm; the black arrow represents inflammation infiltration, edema, hemorrhage, and acinar cell necrosis, and the red arrow represents fibrous hyperplasia and granulation tissue). **(B)** Pancreatic pathological scores from 0 to 5. **(C)** Serum amylase activity. **(D)** Pancreatic lipase activity. Data are expressed as the mean ± SD (*n* = 6). **p < 0.01, ***p < 0.001 compared with the Sham group.

The results showed that the pancreas exhibited intact pancreatic characteristics with normal structures in the Sham and Normal groups. However, the pancreas in the SAP group showed a small amount of inflammation infiltration, edema, hemorrhage, and acinar cell necrosis (black arrow) at 1–3 h, and these pathological changes further deteriorated at 6–48 h. The pancreas of SAP rats were in a self-healing state with massive fibrous hyperplasia and granulation tissue formation (red arrow) at 72–336 h (**Figure 2A**). The pancreatic pathological score in the SAP group was markedly greater than that in the Sham group at different time points (**Figure 2B**). Compared with the Sham group, the activities of serum amylase and pancreatic lipase in the SAP group were evidently elevated at 1–48 h and at 3–24 h, respectively (**Figures 2C, D**). Overall, these results illustrated that the SAP rat presented self-limiting pathological changes from the early injury aggravation (1–48 h) to later continuous repair (72–336 h), indicating that the SAP rat model was successfully established.

### Intestinal Mucosal Barrier Dysfunction Occurred in the Early Stage After SAP Model Construction

The intestinal mucosal barrier function helps maintain intestinal homeostasis (25). As an intestinal metabolite, the serum D-lactate level can reflect the changes in intestinal mucosal permeability (7, 17). The changes in intestinal mucosal barrier function of SAP rats can be determined by observing pathological changes in the small intestines and detecting the serum D-lactate level.

**Figure 3:**
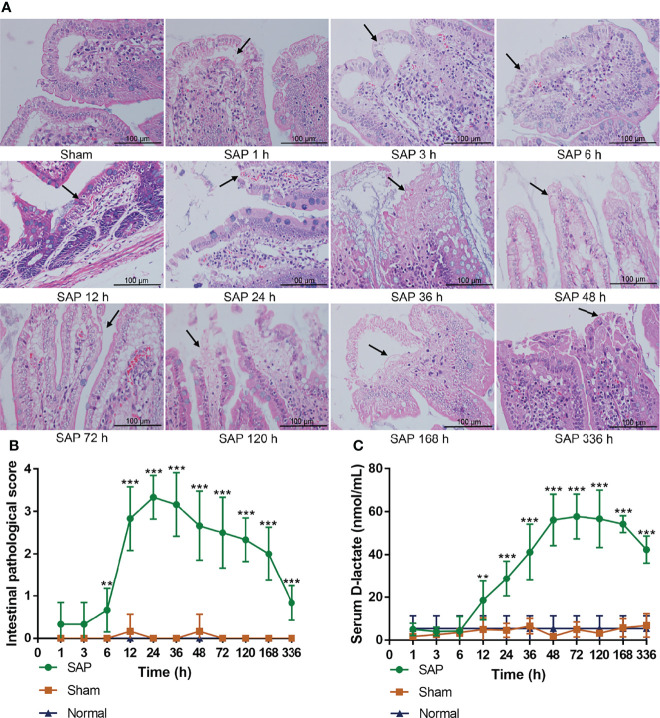
Damage changes in the small intestines at different time points. **(A)** Pathological observation of small intestines (*n* = 3,200 × magnification, scale bar = 100 µm; the black arrow represents intestinal dilatation, epithelial cell shedding, and inflammatory infiltration). **(B)** Intestinal pathological scores from 0 to 4. **(C)** Level of serum D-lactate. Data are expressed as the mean ± SD (*n* = 6). **p < 0.01, ***p < 0.001 compared with the Sham group.

The results showed that the small intestines were structurally intact in the Sham and Normal groups. However, the small intestine in the SAP group showed obvious changes in morphology and structure, such as intestinal dilatation, epithelial cell shedding, and inflammatory infiltration (black arrow) at 6 h, and the tissue damages were further aggravated at 12–48 h, but gradually ameliorated at 48–336 h (**Figure 3A**). The intestinal pathological score in the SAP group was meaningfully higher than that in the Sham group after 12 h, which increased at 6 h, basically kept a higher level at 12–48 h, but gradually decreased at 48–336 h (**Figure 3B**). The serum D-lactate level in the SAP group gradually increased at 12 h, and slightly decreased after maintaining a higher level at 48–120 h (**Figure 3C**). The aforementioned results suggested that the intestinal mucosal barrier function was partially impaired, and the gut permeability was markedly increased in the early stage after SAP model construction.

### Counterbalance of Pro- and Anti-Inflammation Occurred at 24–48 h After SAP Model Construction

Given that inflammation is the main initiator of intestinal injury caused by SAP, we sought to explore the dynamic changes in intestinal pro- and anti-inflammatory cytokine levels to reflect changes in intestinal mucosal immune function during SAP.

**Figure 4:**
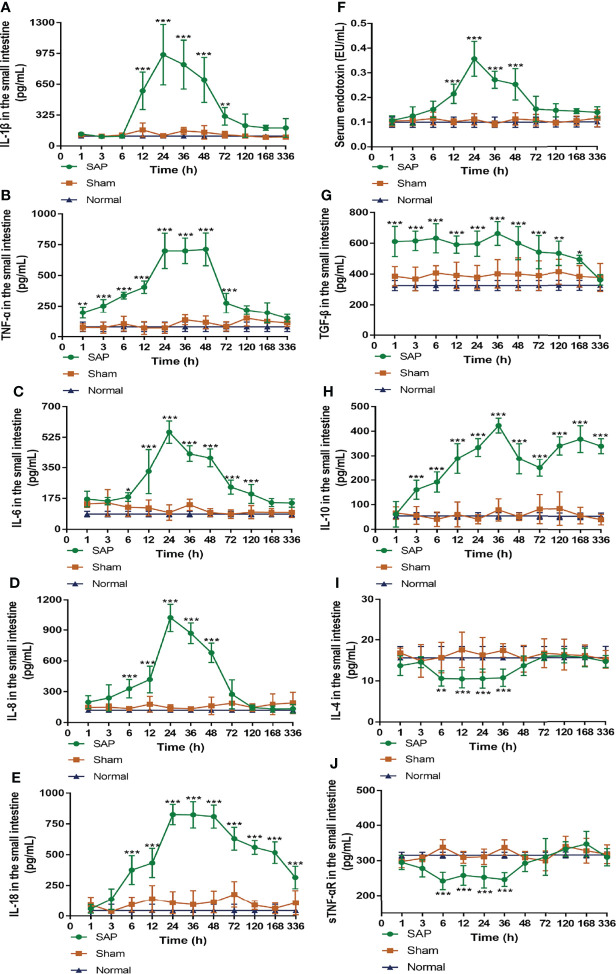
Dynamic changes in pro- and anti-inflammatory cytokines. **(A–E)** Levels of pro-inflammatory cytokines in the small intestines. **(A)** IL-1β. **(B)** TNF-α. **(C)** IL-6. **(D)** IL-8. **(E)** IL-18. **(F)** Serum endotoxin. (G–J) Levels of anti-inflammatory cytokines in the small intestines. **(G)** TGF-β. **(H)** IL-10. **(I)** IL-4. **(J)** sTNF-αR. Data are expressed as the mean ± SD (*n* = 6). *p < 0.05, **p < 0.01, ***p < 0.001 compared with the Sham group.

The detection of intestinal pro-inflammatory cytokines found that, compared with the Sham group, the level of IL-1β in the SAP group increased at 12 h, peaked at 24 h, and hereafter gradually decreased until it dropped to normal levels at 120 h (**Figure 4A**). Compared with the Sham group, the level of TNF-α in the SAP group was slowly elevated at 1–12 h, then quickly increased at 12 h, and kept at a higher level at 24–48 h, which rapidly declined to normal levels at 120 h (**Figure 4B**). Compared with the Sham group, the levels of IL-6, IL-8, and IL-18 in the SAP group considerably increased at 6 h and peaked at 24 h. Then, the levels of IL-6 and IL-8 gradually decreased at 24 h, and the former fell to normal levels at 168 h, while the latter dropped to normal levels at 72 h (**Figures 4C, D**). The level of IL-18 in the SAP group after maintaining a higher level at 24–48 h gradually decreased at 48–336 h, which was still higher than that in the Sham group (**Figure 4E**). Compared with the Sham group, the level of serum endotoxin in the SAP group increased at 12 h, peaked at 24 h, remained at a higher level at 24–48 h, and gradually decreased to normal levels at 72 h (**Figure 4F**).

The results of intestinal anti-inflammatory cytokines showed that, compared with the Sham group, the level of intestinal TGF-β in the SAP group slowly increased at 1 h, remained basically unchanged at 1–48 h, and then slowly declined at 48–168 h until it eventually decreased to normal levels at 336 h (**Figure 4G**). Interestingly, the level of intestinal IL-10 in the SAP group showed the double peaks, which gradually increased at 1–36 h, greatly decreased at 36–72 h, and then gradually increased again at 72–336 h (**Figure 4H**). The levels of intestinal IL-4 and sTNF-αR in the SAP group gradually decreased at 3 h, remained low at 6–36 h, then gradually increased at 36 h until they finally returned to normal levels at 48 h (**Figures 4I, J**).

Taken together, these data demonstrated that the inflammatory response of SAP rats was developed at 1–12 h, during which the pro-inflammatory and anti-inflammatory cytokines gradually increased. Subsequently, the inflammatory cytokine storm was observed at 12–24 h, during which the pro-inflammatory response became dominant. Then, both pro-inflammatory and anti-inflammatory cytokines reached their peak levels at 24–48 h and were counterbalanced by each other. Eventually, a hypo-inflammatory response was dominant after 48 h. As a result, the counterbalance of pro- and anti-inflammation occurred at 24–48 h after SAP model construction.

### Intestinal Innate and Adaptive Immune Switch Occurred at 24–48 h After SAP Model Construction

Innate immune cells in the small intestines activate inflammasome signaling pathways *via* pattern recognition receptors, rapidly initiating inflammatory responses (26, 27). Macrophages and dendritic cells in the small intestines are the main inflammatory cells involved in intestinal innate immune response (28, 29). CD68 and CD103 are regarded as specific markers of macrophages and dendritic cells, respectively (30, 31). An immunohistochemical staining method was used to analyze the dynamic changes in intestinal macrophages and dendritic cells in each group.

**Figure 5:**
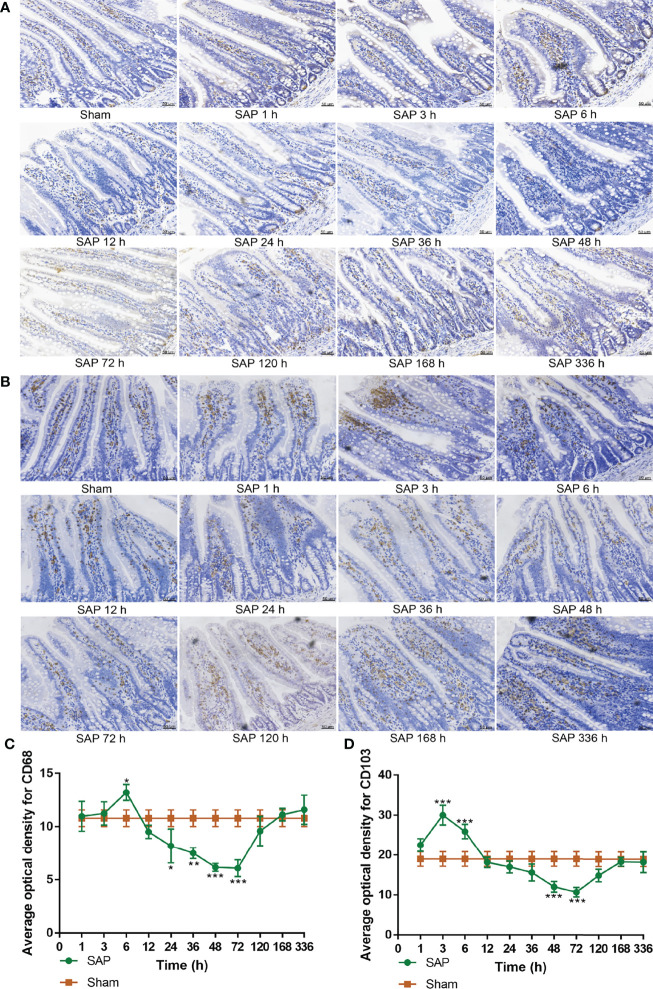
Dynamic changes in intestinal innate immune cells. **(A, B)** Measurement of macrophages and dendritic cells in the small intestines. Representative images of macrophages **(A)** and dendritic cells **(B)** for immunohistological staining (200 × magnification, scale bar = 50 µm). **(C, D)** Average optical density of macrophages and dendritic cells. **(C)** Macrophages. **(D)** Dendritic cells. Data are expressed as the mean ± SD (*n* = 3). *p < 0.05, **p < 0.01, ***p < 0.001 compared with the Sham group.

The results showed that the expression of intestinal CD68 in the SAP group gradually increased at 1–6 h, greatly decreased at 12–72 h, and gradually increased again at 120–336 h compared with the Sham group (**Figures 5A, C**). Compared with the Sham group, the expression of intestinal CD103 in the SAP group gradually increased at 1–3 h, slowly reduced at 6–72 h, and gradually rose again at 120–336 h (**Figures 5B, D**). The above results verified that the number of intestinal innate immune cells increased and the inflammatory response was enhanced at 1–3 h. At 6–72 h, the number of intestinal innate immune cells decreased, resulting in declined cellular immune function and intestinal immune barrier dysfunction. After 120 h, the number of intestinal innate immune cells returned to normal levels.

**Figure 6:**
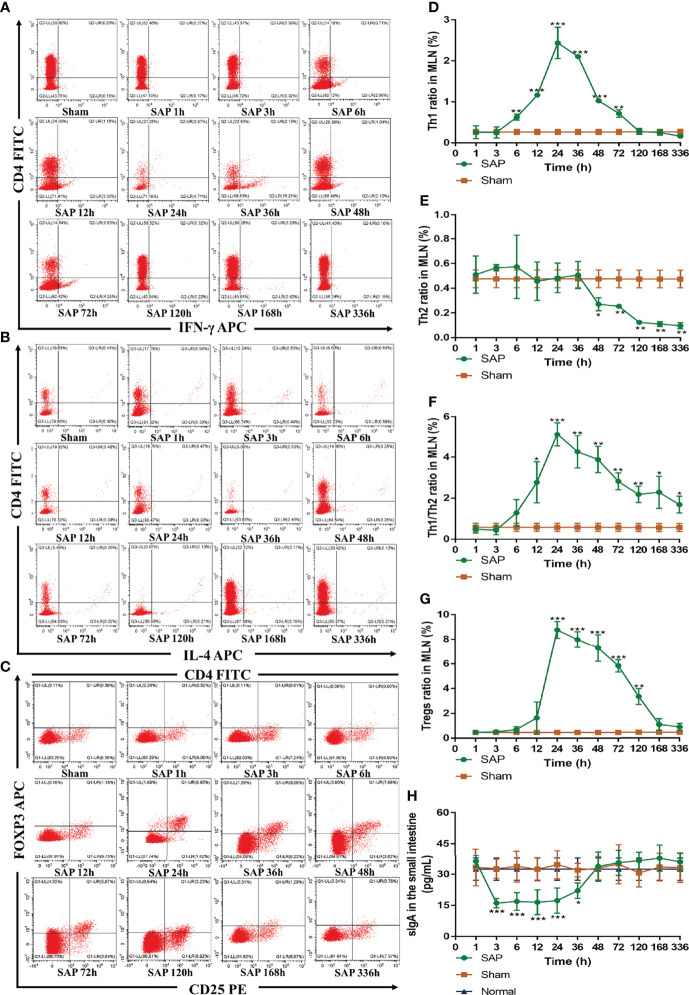
Dynamic changes in intestinal adaptive immune cells. **(A–G)** Detection of T-cell differentiation (Th1 and Th2 cells) and Tregs by flow cytometry in MLN cells. Representative images of Th1 cells **(A)**, Th2 cells **(B)**, and Tregs **(C)** for cytometry results. Quantified analyses of Th1 cells **(D)** and Th2 cells **(E)**. **(F)** Ratio of Th1/Th2 cells. **(G)** Quantified analyses of Tregs. **(H)** Level of sIgA in the small intestines (*n* = 5). Data are expressed as the mean ± SD (*n* = 3). *p < 0.05, **p < 0.01, ***p < 0.001 compared with the Sham group.

Th cells, Tregs, and sIgA are indispensable parts in the adaptive immune response (32). Thus, we further evaluated their changes in SAP rats. We firstly investigated the changes in intestinal Th cells in SAP rats. Th cells are the central cells of the body’s adaptive immune response (32). The expressions of CD4^+^/IFN-γ^+^ and CD4^+^/IL-4^+^ serve to quantify the activations of Th1 cells and Th2 cells, respectively (13). The results revealed that the expression of Th1 cells in the SAP group was significantly higher than that in the Sham group at 6–72 h, which did not significantly change at 1–3 h, gradually increased at 6–24 h, peaked at 24 h, and gradually decreased until it dropped to normal levels at 120 h (**Figures 6A, D**). The expression of Th2 cells in the SAP group was significantly lower than that in the Sham group at 48–336 h. Th2 cells did not obviously change at 1–36 h, but slowly declined at 48–336 h (**Figures 6B, E**). The ratio of Th1/Th2 in the SAP group was significantly higher than that in the Sham group at 12–336 h, which did not significantly change at 1–3 h, gradually elevated at 6–24 h, peaked at 24 h, and then gradually downregulated at 24–336 h (**Figure 6F**). As a result, severely imbalanced Th1/Th2 was observed in SAP rats.

Next, we studied the changes in Tregs in SAP rats. Tregs, which are regarded as suppressive T cells, play a crucial role in maintaining the balance between immune activation and tolerance (13). The expression of CD4^+^/CD25^+^/FOXP3^+^ is identified as the most specific marker of Tregs (13). The results showed that Tregs in the SAP group were obviously higher than those in the Sham group at 24–120 h, which greatly increased at 12 h, slowly declined at 24–48 h after peaking at 24 h, gradually decreased at 48–120 h, until it finally returned to normal levels at 168 h (**Figures 6C, G**).

Finally, we also detected the changes in intestinal sIgA, which is the first line of intestinal mucosa defense and exerts a key role in humeral immunity (33). The results showed that the level of intestinal sIgA in the SAP group was evidently lower than that in the Sham group at 3–36 h, which gradually decreased at 1 h, remained at a lower level at 3–36 h, gradually upregulated at 36 h, and finally returned to normal levels at 48 h (**Figure 6H**). All the above results indicated that intestinal innate and adaptive immune switch occurred at 12–48 h after SAP model construction.

### Confirmation of the Switching Point for Immunosuppression in SAP Rats

Obviously, there were still some differences in the immunoswitching points of changes in various intestinal inflammatory cytokine levels and immune cell numbers. Hence, in further experiments, PA was selected as a second hit to further confirm the immunoswitching point from excessive inflammatory response to immunosuppression in SAP rats.

**Figure 7:**
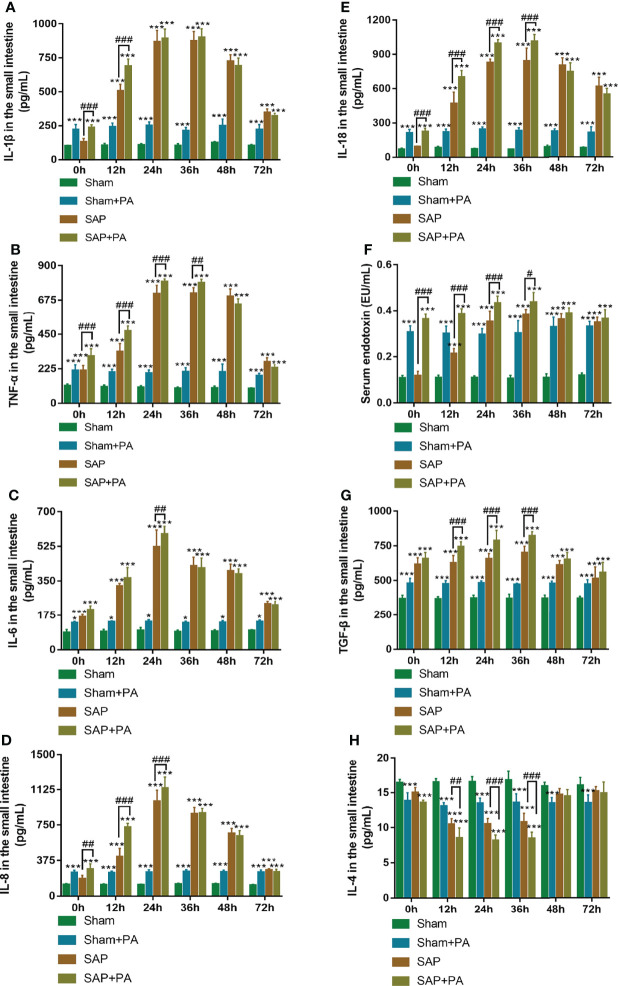
Immune-inflammatory profile with immunosuppressed rats. **(A–F)** Levels of pro-inflammatory cytokines in the small intestines. **(A)** IL-1β. **(B)** TNF-α. **(C)** IL-6. **(D)** IL-8. **(E)** IL-18. **(F)** Serum endotoxin. **(G, H)** Levels of anti-inflammatory cytokines in the small intestines. **(G)** TGF-β. **(H)** IL-4. Data are expressed as the mean ± SD (*n* = 5). *p < 0.05, ***p < 0.001 compared with the Sham group; ^#^*p* < 0.05, ^##^*p* < 0.01, ^###^*p* < 0.001 compared with the SAP group.

The rats were injected with a sublethal dosage of PA (2.0×10^8^ CFU/kg; **Supplemental Table 2**) *via* the tail vein at 0–72 h after Sham or SAP model construction, followed by sacrifice at 2 h post injection (**Supplemental Figures 1A, B**). The detection of pro- and anti-inflammatory cytokines of Sham or SAP rats challenged with or without PA is shown in **Figure 7**. The results showed that the levels of IL-1β, TNF-α, IL-6, IL-8, IL-18, and serum endotoxin significantly increased in Sham + PA, SAP, and SAP + PA groups compared with the Sham group at 0–72 h. The same trend was also found in TGF-β level, while the level of IL-4 presented a trend of first gradually decreasing (0–36 h) and then gradually increasing (48–72 h). Compared with the SAP group, most of the cytokines in the SAP + PA group showed a different degree of increase at 0–36 h, but turned around at 48 h, during which even if rats were challenged with PA, their levels did not increase (**Figures 7A–H**).

**Figure 8:**
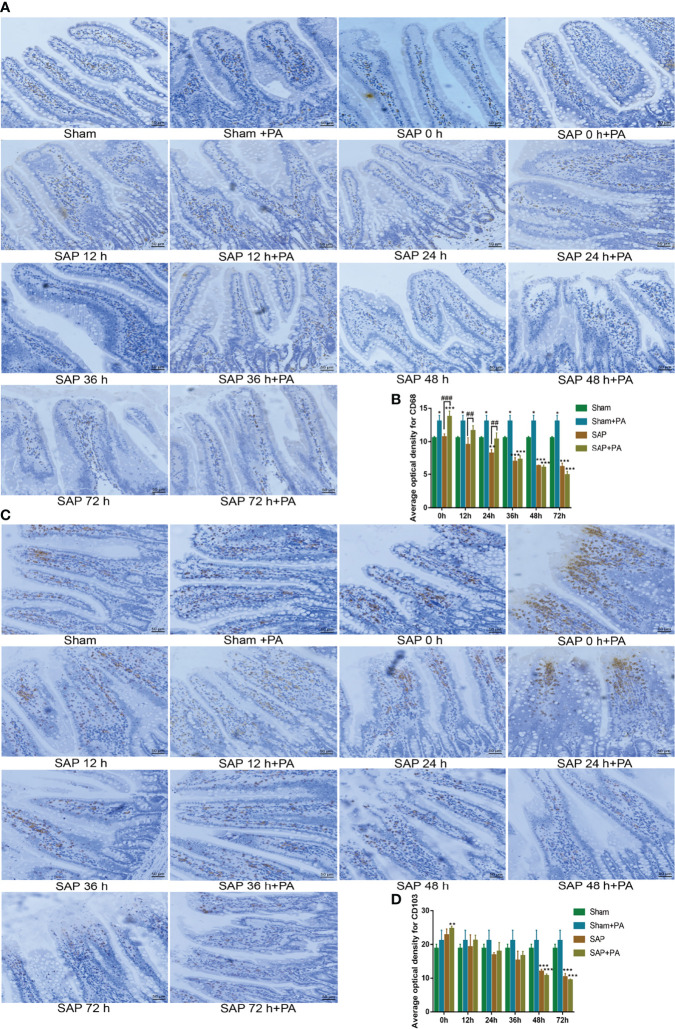
Change of immune cells with immunosuppressed rats. **(A, B)** Measurement of macrophages in the small intestines. **(A)** Representative images of macrophages for immunohistological staining (200 × magnification, scale bar = 50 µm). **(B)** Average optical density of macrophages. **(C, D)** Measurement of dendritic cells in the small intestines. **(C)** Representative images of dendritic cells for immunohistological staining. **(D)** Average optical density of dendritic cells. Data are expressed as the mean ± SD (*n* = 3). *p < 0.05, **p < 0.01, ***p < 0.001 compared with the Sham group; ^##^*p* < 0.01, ^###^*p* < 0.001 compared with the SAP group.

We also analyzed the changes in macrophages and dendritic cells. The results found that after challenge with PA, the number of macrophages in the Sham + PA group and the SAP-0 h + PA group significantly increased. Then, the number of macrophages in the SAP + PA group gradually decreased, which was significantly higher than that in the SAP group at 0–24 h, but with a reversal at 48 h. The changing pattern of dendritic cells was obviously different. Except for the SAP-0 h + PA group, there was no significant increase in other groups. Compared with the Sham group, the number of dendritic cells in the SAP group and the SAP + PA group greatly decreased from 48 h (**Figures 8A–D**). These data confirmed that the rats after challenge with PA showed excessive activation of immune cells and further secretion of cytokines, but the SAP rats changed their immune status into an immunosuppressive one at 48 h, where the immune cells were exhausted, showing a hypo-inflammatory response.

## Discussion

SAP is an acute abdominal disease with a high mortality rate, showing characteristics of immune dysfunction (1–3). Early death due to systemic inflammatory response and multiple organ failure triggered by SAP, and later death as a result of infection caused by immunosuppression are two death peaks in the course of the disease (34). In this study, the SAP rat model was established by a retrograde injection of 3.5% sodium taurocholate into the biliopancreatic duct. The results of pancreatic pathological changes and the activities of serum amylase and pancreatic lipase proved that SAP rats showed self-limited pathological changes from early injury aggravation to later continuous repair, which was consistent with previous reports (35, 36). It was confirmed that 12–24 h and 48–72 h were two death peaks in SAP rats.

The intestine is one of the most vulnerable target organs in SAP (37). The intestinal mucosal barrier dysfunction can promote the progression of systemic inflammatory response and multiple organ failure of SAP patients (38). It was found that intestinal dilatation, epithelial cell shedding, and inflammatory infiltration occurred at 12 h in SAP rats. The severity of SAP patients is closely related to intestinal mucosa permeability (12, 39), and intestinal mucosal permeability is commonly known as the most direct and accurate element that reflects intestinal mucosal barrier function (40, 41). D-lactate, which is a kind of the intestinal metabolite, enters the blood circulation and significantly increases when the intestinal mucosal barrier function is seriously damaged (42). In this study, the serum D-lactate level in SAP rats gradually increased at 12 h, but slightly reduced after maintaining a higher level at 48–120 h. Interestingly, this result conformed to the time point at which early death occurred at 12 h after SAP model construction, suggesting that the early death of SAP rats could be related to intestinal mucosal barrier dysfunction caused by increased intestinal mucosal permeability. Therefore, the severity of SAP could be suppressed by improving the intestinal mucosal barrier function, which is of great significance for the development of new therapeutic options for SAP.

The intestinal immune system is considered as the most important line of defense against invasion of enteric microorganisms and exerts a vital role in the development of SAP (32, 43). There is a dynamic and complex change rule from excessive inflammatory response to immunosuppression in the SAP immune system (44). However, the possible reason why the immunoswitching point from excessive inflammatory response to immunosuppression was different in different laboratories was the lack of clinically validated indicators and complete assessment for monitoring the immune function of SAP. In the early stage of SAP, locally damaged pancreatic tissue and inflammatory cells cause damage to the intestinal mucosal barrier function, translocation of bacteria and endotoxins, and further activation of intestinal epithelial cells, macrophages, and other innate immune cells, leading to the release of a large number of cytokines and eventually triggering inflammatory cascades (45). IL-1β, TNF-α, IL-6, IL-8, and IL-18 are widely reported pro-inflammatory cytokines that are key indicators for the early prediction and diagnosis of pancreatitis (46–48). Endotoxin is a component of the cell wall of Gram-negative bacteria, and large amounts of endotoxin into the blood can cause endotoxemia and even trigger the systemic inflammation response (49, 50). TGF-β, IL-10, IL-4, and sTNF-αR are well-known anti-inflammatory cytokines (24). In general, we found that intestinal pro-inflammatory cytokines (including IL-1β, TNF-α, IL-6, IL-8, IL-18, and serum endotoxin) and anti-inflammatory cytokines (including TGF-β and IL-10) in SAP rats were at a higher level at 12 h after model construction, which was consistent with the time point of the early death peak of SAP rats, suggesting that the early death of SAP rats was due to an inflammatory storm caused by the excessive release of pro-inflammatory cytokines and anti-inflammatory cytokines. The results were in line with a previous report (34). The possible reasons for the double peaks of the intestinal IL-10 level in SAP rats were as follows: the inflammatory response was enhanced at 1–36 h, the innate immune cells that released IL-10 were damaged at 48 h, and the immune function of the rats gradually recovered after 72 h. Interestingly, the levels of intestinal IL-4 and sTNF-αR in SAP rats were opposite to those of pro-inflammatory cytokines, showing a trend of first decreasing and then increasing, which could be closely related to the early suppression of Th2 cells and the over-secretion of competing sTNF-αR by TNF-α. In summary, we believed that although some anti-inflammatory cytokines were also elevated in the early stage of SAP, pro-inflammation response was dominant. In the later stage, all of the pro-inflammatory and some of the anti-inflammatory cytokines were relatively reduced as a result of the death of immune cells, resulting in a hypo-inflammatory response status in SAP rats.

The immune cells that secrete inflammatory cytokines also play an important role during SAP. Excessive activation of inflammatory immune cells results in the cascade release of inflammatory cytokines (51). Intestinal mucosal immune function mainly depends on the innate and adaptive immunity of intestinal mucosal immune cells, and their role in the development of SAP has been widely discussed (52). The innate immune system is the first line of defense against intestinal pathogen infections (43). Macrophages are the main inflammatory cells involved in the pathogenesis of SAP, secreting inflammatory cytokines such as IL-1β and TNF-α, and the number and activation of macrophages determine the severity of SAP (28, 53, 54). Dendritic cells are potent antigen-presenting cells that drive both adaptive and innate immunity, secreting inflammatory cytokines including TNF-α and IL-6 (55, 56). It was hypothesized that the increase in the number of intestinal macrophages and dendritic cells at 1–3 h could be related to the activation of pancreatic enzymes in pancreatic cells, which led to a large number of immune cells activated and chemoattracted to the intestine, further activating the intestinal innate immune cells, and ultimately resulting in an enhanced inflammatory response. However, the number of intestinal macrophages and dendritic cells was decreased at 6–72 h, when the intestinal innate cells could trigger pyroptosis, causing a more intense inflammatory cascade with massive death of immune cells (57). After 120 h, the number of intestinal macrophages and dendritic cells gradually returned to normal levels, and the SAP rats were in a self-healing state.

Th cells, Tregs, and sIgA are indispensable parts in the adaptive immune response (32). The ratio of various subtypes of T cells can reflect the immune status of SAP patients (58). Th1 cells mainly produce pro-inflammatory cytokines to mediate cellular immunity, while Th2 cells mainly produce anti-inflammatory cytokines to regulate humoral immunity (59, 60). It was found that the ratio of Th1 cells in SAP rats was significantly higher than that in Sham rats at 6–72 h, while the ratio of Th2 cells in SAP rats was obviously lower than that in Sham rats at 48–336 h, suggesting that Th1 cells participated in the early inflammatory response of SAP by releasing pro-inflammatory cytokines and Th2 cells mainly secreted anti-inflammatory cytokines to counter excessive inflammatory response. Th1 cells inhibit Th2 cells after their activation by antigens to induce Th1/Th2 cytokine dynamic imbalance, resulting in a series of inflammatory response, aggravating intestinal injury, and further triggering the systemic inflammatory response and other serious consequences in SAP patients (61). The balance of Th1/Th2 is crucial for maintaining homeostasis in the body (62, 63). Flow cytometry results from MLNs showed that the ratio of Th1/Th2 in SAP rats was significantly higher than that of Sham rats at 12–336 h, indicating that Th1/Th2 is seriously imbalanced in SAP rats; inflammatory responses leaned towards pro-inflammatory ones, which was inconsistent with the changes in intestinal inflammatory cytokines. The results suggested that the changes in cytokines could also be affected by other intestinal immune cells such as Peyer’s patches, which will be our future research direction. Tregs exert immunosuppressive effects by inhibiting INF-γ secretion and promoting IL-4, IL-10, and TGF-β secretion (64, 65). A study has proved that Tregs were decreased and inversely associated with the serum concentration of TNF-α in SAP mice (66). In this study, Tregs in SAP rats were obviously higher than those in Sham rats at 24–120 h, which was positively correlated with intestinal TGF-β and IL-10 levels. SIgA is a main immunoglobulin protein on the intestinal mucosal surface secreted by B lymphocytes after differentiating into plasma cells *via* antigen stimulation and activation, and its expression is negatively correlated with the severity of SAP (67, 68). Clinical research found that the sIgA level was clearly decreased in the peripheral blood of SAP patients (69). We also found that the intestinal sIgA level in SAP rats was evidently lower than that in Sham rats at 3–36 h, when the function of B lymphocyte in SAP rats was severely destroyed. The result was supported by a previous study (32).

The above results elucidated that the immunoswitching period from excessive inflammatory response to immunosuppression occurred at 24–48 h after SAP model construction, but there were still some differences in the immunoswitching points of changes in various intestinal inflammatory cytokine levels and immune cell numbers. In the stage of immunosuppression, the body’s ability to release inflammatory cytokines decreases and the re-stimulation will not cause an increase in pro-inflammatory cytokines, which is characterized by low immune response or no response (70, 71). Immunosuppression mainly manifests as decreased immune cell numbers and functions, including macrophage inactivation and decreased antigen presentation ability (72). We found that the levels of intestinal inflammatory cytokines and the numbers of immune cells turned around at 48 h, which could be related to the immunosuppression of SAP rats, showing that 48 h after SAP model construction was the immunoswitching point from excessive inflammatory response to immunosuppression in SAP rats.

This study systematically explored the immunoswitching point from excessive inflammatory response to immunosuppression in SAP rats. Although this study has indicated that 24 h after SAP model construction was the immunoswitching point (73), it only evaluated SAP immune status from the changes in pro-inflammatory cytokines. In a sepsis-immunosuppressed mouse model, only pro-inflammatory cytokine and bacterial clearance changes were measured. Therefore, we believe that it is more meaningful to combine the changes in anti-inflammatory cytokine levels and immune cell numbers to assess the immune function of SAP. Importantly, therapeutics targeting immunoswitching point hold significant potential to reverse sepsis-induced immunosuppression and preserve host immunity against primary and secondary infections (74). The physiological and pathological process of SAP is similar to that of sepsis, and it is speculated that this special phenomenon also exists in the SAP model. Although clinical studies on the immunoswitching point from excessive inflammation to immunosuppression are currently lacking, researchers have confirmed that inflammatory cytokines increased in the early stage and decreased in the later stage during SAP. The results of this study will lay a solid foundation for the follow-up assessment of the course of SAP and the search for the optimal timing of immunomodulatory drugs.

## Data Availability Statement

The original contributions presented in the study are included in the article/**Supplementary Material**. Further inquiries can be directed to the corresponding authors.

## Ethics Statement

The animal study was reviewed and approved by the Animal Ethics Committee of Southwest Medical University (Luzhou, China).

## Author Contributions

YX and YeZ conceived, designed, and supervised the study. QZ, LH, YuZ, and LW conducted experiments. QZ and LH performed data statistics and analysis, and wrote the manuscript. XW and YX modified the manuscript. GQ and ML revised the article for intellectual content. All authors contributed to the article and approved the submitted version.

## Funding

This research was supported by the Project of National Natural Science Foundation of China (Nos. 81873067 and 81102868).

## Conflict of Interest

The authors declare that the research was conducted in the absence of any commercial or financial relationships that could be construed as a potential conflict of interest.

## Publisher’s Note

All claims expressed in this article are solely those of the authors and do not necessarily represent those of their affiliated organizations, or those of the publisher, the editors and the reviewers. Any product that may be evaluated in this article, or claim that may be made by its manufacturer, is not guaranteed or endorsed by the publisher.
