# Supplementary material for: Dynamic Monitoring of Immunoinflammatory Response Identifies Immunoswitching Characteristics of Severe Acute Pancreatitis in Rats
Source: Front Immunol. 2022 May 19;13:876168. doi: 10.3389/fimmu.2022.876168 (PMC9160235; doi:10.3389/fimmu.2022.876168)
Supplement: Supplementary file 1 [file DataSheet_1.pdf]

Supplementary Material

1 Supplementary Methods

1.1 Screening of a Sublethal Dose of PA for Rats

Different doses of PA (1.0×10^8, 2.0×10^8, 4.0×10^8, 8.0×10^8, 1.0×10^9, 1.0×10^{10} CFU/kg) were injected into the tail vein of rats in Sham group. The mortality of each group was observed for 7 days to obtain a sublethal dose of PA.

1.2 Sensitivity Observation of Rats With PA Challenge at Different Time Points

The rats were randomly divided into Normal group and challenged with PA group. The Normal group was not treated. The challenged with PA group was injected with a sublethal dose of PA (2.0×10^8CFU/kg) into the tail vein, and the challenged with PA group was divided into 5 subgroups, which were sacrificed at 0 h, 2 h, 4 h, 6 h, and 12 h after PA injection. A best sampling time was determine by ELISA method to detect the levels of IL-1β and TNF-α in the small intestines.

2 Supplementary Results

2.1 Supplementary Tables

Table 1. The 14-day cumulative survival rate of SAP rats.

Time	SAP（N = 110）		Sham（N = 66）	
	deaths/totol	cumulative survival rate (%)	deaths/totol	cumulative survival rate (%)
1h	0/110	100.00%	0/66	100.00%
3h	0/100	100.00%	0/60	100.00%
6h	0/90	100.00%	0/54	100.00%
12h	5/80	93.75%	0/48	100.00%
24h	6/70	85.71%	0/42	100.00%
36h	1/60	84.28%	0/36	100.00%
48h	2/50	80.91%	0/30	100.00%
72h	2/40	76.86%	0/24	100.00%
120h	0/30	76.86%	0/18	100.00%
168h	1/20	73.02%	0/12	100.00%
336h	1/10	65.72%	0/6	100.00%

For survival analysis, the cumulative survival rate in each group was recorded for 14 days after Sham and SAP construction.

Table 2. 7-day mortality of rats challenged with PA at different dosages (CFU/kg).

PA dosage	Sham	1.0×10^8	2.0×10^8	4.0×10^8	8.0×10^8	1.0×10^9	1.0×10^{10}
deaths/total	0/8	0/8	0/8	4/8	7/8	8/8	8/8
Mortality (%)	0%	0%	0%	50%	87.50%	100%	100%

The mortality of each group was observed for 7 days to obtain a sublethal dose of PA in rat model.

2.2 Supplementary Figure

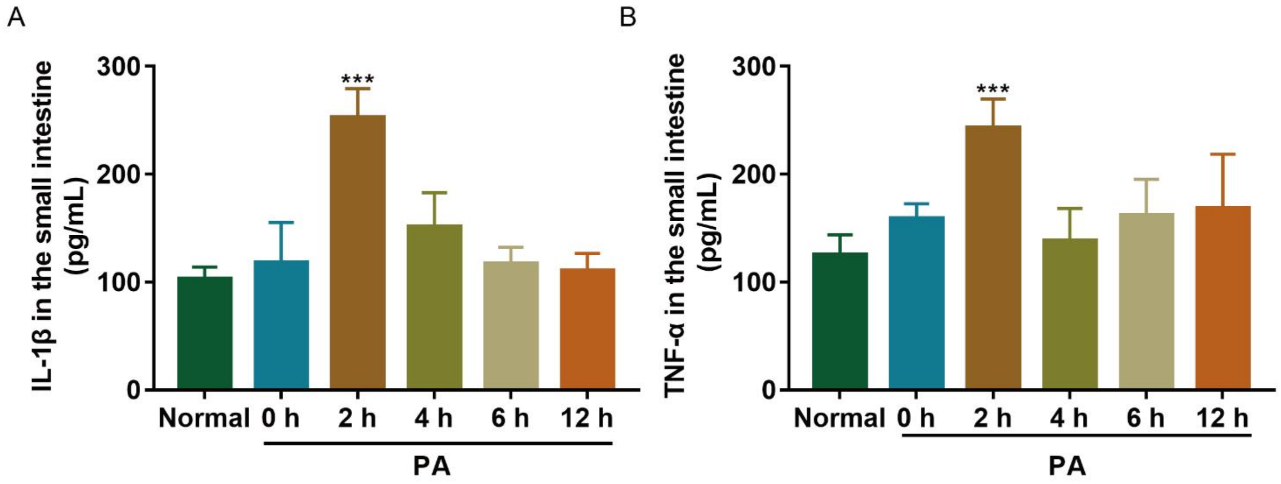

Figure 1 | Determination of the best timing point of sampling. (A) Level of IL-1β in the small intestine. (B) Levels of TNF-α in the small intestine. Differences are assessed by one-ANOVA and expressed as the mean ± SD (n = 5). *** $p < 0.001$ compared with the Normal group.
